# Accumulation and Toxicity of Copper Oxide Engineered Nanoparticles in a Marine Mussel

**DOI:** 10.3390/nano4030535

**Published:** 2014-06-27

**Authors:** Shannon K. Hanna, Robert J. Miller, Hunter S. Lenihan

**Affiliations:** 1 Bren School of Environmental Science and Management, University of California, Santa Barbara, CA 93106-5131, USA; E-Mail: Lenihan@bren.ucsb.edu; 2 Marine Science Institute, University of California, Santa Barbara, CA 93106-6150, USA; E-Mail: miller@msi.ucsb.edu

**Keywords:** CuO, nanomaterials, mussel, ecotoxicology

## Abstract

Cu is an essential trace element but can be highly toxic to aquatic organisms at elevated concentrations. Greater use of CuO engineered nanoparticles (ENPs) may lead to increased concentrations of CuO ENPs in aquatic environments causing potential ecological injury. We examined the toxicity of CuO ENPs to marine mussels and the influence of mussels on the fate and transport of CuO ENPs. We exposed marine mussels to 1, 2, or 3 mg L^−1^ CuO ENPs for four weeks, and measured clearance rate, rejection, excretion and accumulation of Cu, and mussel shell growth. Mussel clearance rate was 48% less, and growth was 68% less, in mussels exposed to 3 mg L^−1^ than in control animals. Previous studies show 100% mortality at 1 mg Cu L^−1^, suggesting that CuO ENPs are much less toxic than ionic Cu, probably due to the slow dissolution rate of the ENPs. Mussels rejected and excreted CuO ENPs in biodeposits containing as much as 110 mg Cu g^−1^, suggesting the potential for magnification in sediments. Mussels exposed to 3 mg L^−1^ CuO ENPs accumulated 79.14 ± 12.46 μg Cu g^−1^ dry weight, which was 60 times more Cu than in control animals. Our results suggest that mussels have the potential to influence the fate and transport of CuO ENPs and potentially cause magnification of CuO ENPs in mussel bed communities, creating a significant source of Cu to marine benthos.

## 1. Introduction

CuO engineered nanoparticles (ENPs) are used in industrial applications as a catalyst for carbon monoxide oxidation and a component of heat transfer fluids [1,2]. Their antimicrobial [3] and antifouling properties [4] make CuO ENPs useful in soaps and antifouling paints and coatings for boat hulls and other submerged surfaces, suggesting that release into the environment is likely. Increased production and use will lead to greater environmental release of CuO ENPs, which is of concern because of the lack of information regarding their environmental safety. Although Cu is an essential metal, it is extremely toxic at elevated concentrations and negatively impacts mussels [5,6,7,8,9,10,11,12], fish [13,14,15], and crustaceans [16,17] at concentrations as low as 4 μg L^−1^ [6,9]. Mechanisms of toxicity include interference with osmoregulation due to enzyme inhibition [15,18,19], decreased immune function [7], and decreased respiration [20]. Addition of CuO ENPs to the environment may have negative impacts on marine organisms and ecosystems. However, little is known about the toxicity of CuO ENPs in the aquatic environment. CuO ENPs do not readily dissolve in aqueous media [21,22], suggesting that they may be less bioavailable than Cu salts but more bioavailable than micro-sized CuO. Indeed, CuO ENPs are much more toxic than micro-sized CuO: they are 15 times more toxic to microalgae [23], 60 times more toxic to yeast [24], and 10–20 times more toxic to *Tetrahymena* [22] than conventional CuO material. Although CuO ENPs are more toxic and release more ions than micro-sized CuO, dissolution is minimal and the majority of toxicity caused by CuO ENPs is due to the particle and not the ion [25,26], suggesting a nano-specific toxic effect.

Understanding the fate and transport of CuO ENPs in addition to toxicity will allow us to predict the behavior and impacts of CuO ENPs in environmental systems. Suspension-feeding mussels are likely to alter these processes, as they remove suspended material from the water column, can accumulate portions of it, and concentrate the remaining material in nutrient-rich biodeposits [27]. Individual mussels can filter more than a liter of water per hour [28] and therefore have the potential to consume large amounts of water-borne contaminants including ENPs. Mussels are considered foundation species because they have substantial influence on the species composition and trophic structure of marine communities by modulating fundamental ecosystem processes, creating biogenic habitat, and stabilizing environmental conditions [29]. For example, mussel populations create three-dimensional reefs or hummocks that reduce local hydrodynamic force and desiccation stress but also trap nutrient-rich sediment, factors that enhance the abundance and species diversity of many coastal invertebrate organisms [30,31]. Thus, negative effects of ENPs on mussels could have manifold negative impacts on many other organisms that rely on mussels and their habitat. Their ecological importance, combined with their high potential for exposure as suspension feeders processing large volumes of water, makes mussels a key species for marine toxicity studies.

Here we test the accumulation and toxicity of 1–3 mg L^−1^ CuO ENPs during a month-long exposure to the marine mussel, *Mytilus galloprovincialis*. Mussels exposed to dissolved Cu decrease filtration and clearance rates [5,10,11], accumulate Cu in tissues during exposure [5,11,32,33] with higher concentrations found in the viscera than gills [12], and decrease growth rate [5,10,11]. However, CuO ENPs dissolve slowly in aqueous media [21,22,34], suggesting that the majority of exposure will be via particulate CuO ENPs. We tested the hypotheses that mussels exposed to CuO ENPs will (1) decrease clearance rate; (2) deposit high concentrations of Cu in biodeposits; (3) accumulate Cu throughout the experiment; (4) and decrease growth rate. We test these hypotheses by using linear regression models to quantitatively describe the exposure dose and time dependence of clearance rate, Cu accumulation, Cu deposition and growth, which provide information useful for assaying the risk of this emerging contaminant to marine ecosystems.

## 2. Results

**Figure 1:**
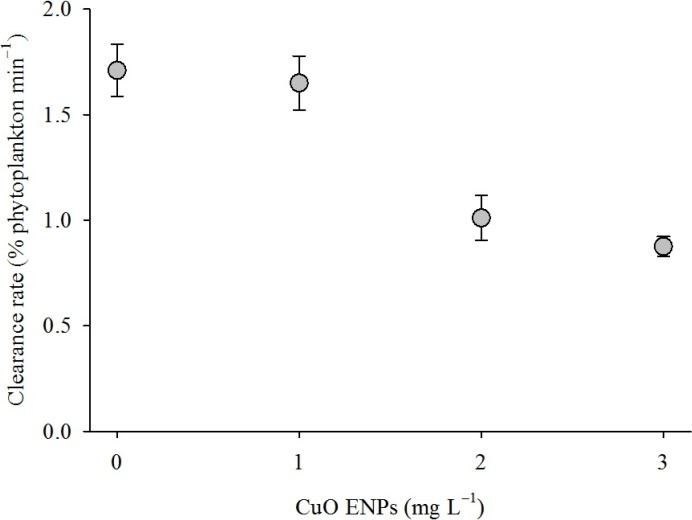
Feeding rate of mussels exposed to CuO engineered nanoparticles (ENPs). Mean feeding rate for mussels exposed to CuO ENPs for four weeks. Feeding rate decreased with increasing CuO ENP concentration (ordinary least squares (OLS): Feeding = 1.99 × 10^−2^ − 3.1 × 10^−^^3^ (Concentration) − 8 × 10^−^^4^ (Time), *r*^2^ = 0.37). Error bars are one standard error of the mean.

CuO ENPs in stock suspensions of 100 mg L^−1^ aggregated to a mean diameter of 723 ± 21 nm immediately after sonication. Mussel clearance rate declined as CuO ENP exposure concentration increased (Figure 1). Clearance rate depended on CuO ENP concentration (Table 1, ordinary least squares (OLS): *t*_2, 75_ = 6.38, *p* < 0.0001) but not exposure time (OLS: *t*_2, 75_ = 1.76, *p* > 0.05). Clearance rate declined by approximately 0.31% ± 0.05% min^−1^ for every 1 mg CuO ENPs L^−1^ (OLS: *r*^2^ = 0.37, *p* < 0.0001), from 1.71 ± 0.12% of phytoplankton min^−1^ for animals in the control group to 0.90 ± 0.04% min^−1^ for mussels exposed to 3 mg L^−1^.

**Figure 2:**
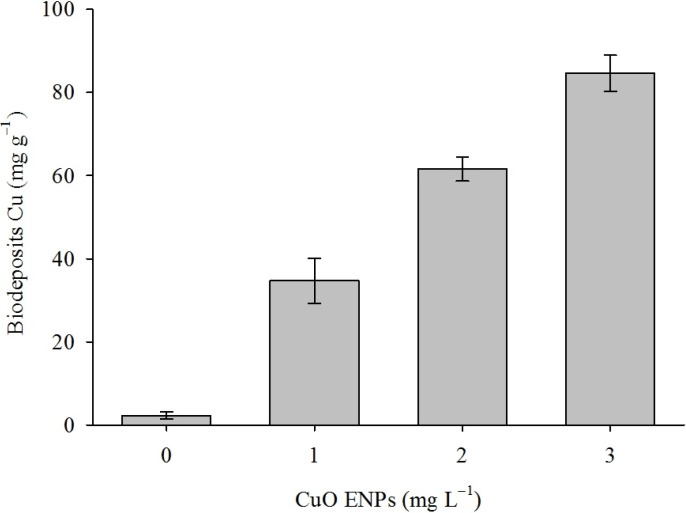
Excretion of Cu in mussels exposed to CuO ENPs. Mean Cu concentration in biodeposits collected from mussels exposed to CuO ENPs for four weeks. Cu concentration in biodeposits increased with increasing CuO ENP concentration (OLS: Excretion = 0.83 + 27.48 (Concentration) + 1.47 (Time), *r*^2^ = 0.85). Error bars are one standard error of the mean.

Mussels exposed to higher concentrations of CuO ENPs excreted biodeposits containing greater amounts of Cu (Figure 2). Cu concentration in biodeposits increased as a function of CuO ENP concentration but not exposure time (Table 1, OLS: *r*^2^ = 0.85, *p* < 0.0001). Biodeposits contained approximately 27,000 times the CuO ENP exposure concentration, reaching as much as 110 mg Cu g^−1^ biodeposits in mussels exposed to 3 mg L^−1^.

**Figure 3:**
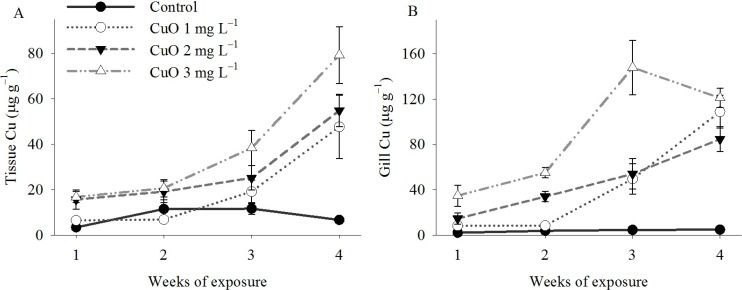
Bioaccumulation of Cu in mussels exposed to CuO ENPs. Mean Cu concentration in (**A**) tissue and (**B**) gill of mussels exposed to CuO ENPs for four weeks. The impact of CuO ENPs on Cu concentration in tissue and gill depended on exposure time (OLS: Tissue Cu = 0.78 − 4.31 (Concentration) + 3.26 (Time) + 5.72 (Concentration × Time), *r*^2^ = 0.72; OLS: Gill Cu = −17.01 + 3.17 (Concentration) + 9.61 (Time) + 9.15 (Concentration × Time), *r*^2^ = 0.74). Error bars are standard error of the mean.

Mussels accumulated greater concentrations of Cu in gill and the remaining tissue (all other tissue other than gill) when exposed to higher concentrations of CuO ENPs (Figure 3A,B). The impact of CuO ENP concentration on bioaccumulation of Cu varied as a function of exposure time in both gill (Table 1, OLS: *r*^2^ = 0.74, *p* < 0.0001) and remaining tissue (OLS: *r*^2^ = 0.72, *p* < 0.0001). Cu in gill from control animals was 2.23 ± 0.1 μg g^−1^ in week one and 4.86 ± 0.5 μg g^−1^ in week four, while Cu concentration in gill from mussels exposed to 3 mg L^−1^ was 34.89 ± 9.4 μg g^−1^ in week one and 121.06 ± 8.4 μg g^−1^ in week four. In the remaining tissue, Cu concentration was 3.43 ± 0.1 μg g^−1^ in week and 6.69 ± 1.3 μg g^−1^ in week four in control mussels but increased from 16.78 ± 2.5 μg g^−1^ in week one to 79.14 ± 12.5 μg g^−1^ by week four in mussels exposed to 3 mg L^−1^ CuO ENPs.

**Figure 4:**
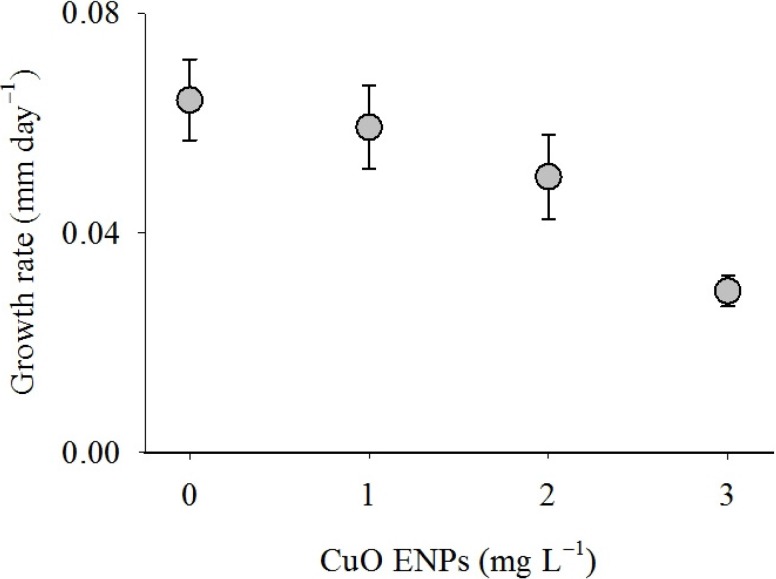
Growth rate of mussels exposed to CuO ENPs. Mean growth rate for mussels in the control group and those exposed to CuO ENPs for four weeks. The impact of CuO ENPs on growth rate depended on exposure time (OLS: Growth = 3.16 × 10^−2^ + 1.39 × 10^−2^ (Concentration) + 1.03 × 10^−2^ (Time) − 6.8 × 10^−^^3^ (Concentration × Time), *r*^2^ = 0.20). Error bars are one standard error of the mean.

Mussel growth generally decreased as CuO ENP concentration increased (Figure 4). The impact of CuO ENPs on growth rate of mussels varied significantly as a function of exposure time (Table 1, OLS: *r*^2^ = 0.20, *p* < 0.01). Mean growth of mussels in control groups was 1.93 ± 0.2 mm during the experiment, while mussels exposed to 3 mg L^−1^ grew less than half as much, 0.82 ± 0.1 mm. Daily growth rate increased for the control group during the experiment but decreased for mussels exposed to 3 mg L^−1^. By the end of the first week of the experiment, mussel growth rate was approximately 0.02 mm/day for control animals and 0.06 mm/day for mussels exposed to 3 mg L^−1^. By the end of the experiment growth rate was 0.06 mm/day for control animals and 0.03 mm/day for mussels exposed to 3 mg L^−1^.

## 3. Discussion

Cu is toxic to mussels at low concentrations [5,6,7,8,9,10,11,12] and mussels accumulate metals when exposed to metal oxide ENPs [35,36,37]. Accordingly, we predicted that mussels exposed to CuO ENPs would filter water less efficiently, deposit CuO ENPs in highly concentrated feces and pseudofeces, accumulate Cu in their tissues, and grow slowly. After exposing mussels to CuO ENPs for four weeks, these predictions were largely supported: we found a decrease in clearance rate, increases in Cu content in biodeposits and tissues, and a decrease in growth rate. Cu content in tissue and growth were impacted by exposure time but clearance rate and Cu in biodeposits were not. These results suggest that as mussels accumulated more Cu in tissues, growth rate decreased but clearance rate was influenced by environmental concentrations of CuO ENPs and not tissue accumulation.

Cu, like many other transition metals, is an essential trace element but it is also highly toxic at elevated levels. *Mytilus edulis* decreased respiration rate by almost 70% when exposed to Cu at 1 mg L^−1^ for two hours, and 100% of mussels died after exposure to 0.5 mg L^−1^ for one week [38]. We exposed mussels to concentrations of CuO ENPs that were six times higher for four times as long, and saw no significant mortality during our study, suggesting that CuO ENPs are less toxic to mussels than Cu ions. CuO ENPs dissolve very slowly in natural matrices; e.g., approximately 2% of CuO ENPs dissolve in growth media within 48 hours of preparing the suspension [21,22]. For example, in a prior study we documented that the same form of CuO ENPs used in this experiment dissolved very slowly in seawater, with only ~1% dissolving in 90 days [26]. This rate implies that mussels in our 3 mg L^−1^ exposure group would have encountered Cu ion concentrations of approximately 0.06 mg L^−1^. However, CuO ENPs dissolve faster in low pH environments [39] such as the mussel gut, which would substantially increase Cu ion exposure. Mussels exposed to Cu concentrations of 0.1 mg L^−1^ did not show increased mortality compared with controls, and 0.2 mg L^−1^ had no impact on respiration rate compared with controls [38]. *Perna perna*, another marine mollusc, exhibited decreased respiration rates but not clearance rates when exposed to Cu at 0.05 mg L^−1^ for 24 days [40]; however, clearance rates were measured after Cu exposure and not in the presence of Cu. *Perna viridis* exposed to 0.05 mg L^−1^ for three months decreased clearance rate in the presence of Cu [5]. Similar to these studies with Cu salts, our results suggest that environmental CuO ENP concentrations, and not Cu accumulation, impacts clearance rate. Current estimates of environmental concentrations of Cu ENPs are very low, with ranges estimating from approximately 0.1 to 0.7 μg L^−1^ [41], however, mussels frequently occupy coastal marine “hot spots” for pollution therefore they are at risk of being exposed to higher concentrations of Cu as well as other ENPs.

Metal accumulation is well documented in mussels but the dynamics of metal ENP uptake and accumulation are poorly understood. Mussels are inefficient at capturing particles <1 μm [42], therefore, they would probably not filter ENPs as readily as larger particles. Nevertheless, mussels accumulate various ENPs including ZnO [35,37], Au [43,44], polystyrene beads [42], carbon black [45], CeO_2_ [35,36],and SiO_2_ [46]. ENP aggregation may facilitate capture, and ENPs have longer gut retention times than larger particles, suggesting that they are transported to the digestive gland [42]. While our measurements indicate that ENPs aggregated, aggregates were <1 μm in diameter. Still, Cu increased in tissue over the duration of our experiment with the largest increase between week three and four in all groups exposed to CuO ENPs. Cu accumulation in our experiment was probably due, at least partly, to dissolved Cu, as mussels exposed to much lower concentrations had similar body burdens. For example, *M. edulis* exposed to 0.01 mg L^−1^ Cu for 18 months accumulated approximately 65 μg g^−1^ wet tissue [47]. *Perna perna* accumulated up to 45 μg g^−1^ dry weight when exposed to 0.05 mg L^−1^ for 24 days [40]. These results are similar to mussels in our 3 mg L^−1^ exposure treatment, which accumulated approximately 80 μg Cu g^−1^ dry tissue after four weeks. If mussels accumulate the ENPs in addition to the metal, this poses the risk of trophic transfer of these contaminants. A number of different marine organisms accumulate ionic copper via trophic transfer [48,49,50]. Information about accumulation of ENPs is sparse, but rotifers have been shown to accumulate quantum dots by consuming exposed protozoans [51]. Additionally, CeO_2_ ENPs have been shown to sorb to live phytoplankton within <1 h after mixing and are most likely accumulated in mussels via consumption of phytoplankton [36].

Suspension-feeding organisms can influence the transport of ENPs in the environment by ingesting and excreting them onto the seafloor, making them available in high concentrations to infaunal and other benthic species living within or near mussel beds. Our results indicate that mussels can concentrate 3 mg L^−1^ of ENPs in suspension by >27,000 times into biodeposits containing >100 mg Cu g^−1^ that are deposited on the seafloor. Similarly, mussels exposed to ZnO and CeO_2_ ENPs excreted biodeposits containing >3000 times the exposure concentrations [35]. This is of concern ecologically considering that mussel bed communities typically harbor abundant and diverse communities, especially deposit feeding invertebrates [52,53,54], which consume sediment and would therefore be exposed internally to elevated Cu concentrations. Much lower concentrations of ENPs in sediment are toxic to infaunal organisms [26,55,56]. This suggests that CuO ENPs would have toxic effects on mussel bed and surrounding soft-sediment communities even if mussel survival were unaffected by low concentrations in the water column.

**Table 1:** Multiple OLS regressions examining the impact of CuO ENP exposure on feeding, Cu excretion, accumulation, and growth.

	Feeding × 10^2^	Excretion/Rejection	Bioaccumulation (Tissue)	Bioaccumulation (Gill)	Growth × 10^2^
**Intercept**	1.99 (0.15) ***	0.83 (5.3)	0.78 (7.15)	−17.01 (14.85)	3.16 (1.34) *
**[CuO]**	−0.31 (0.05) ***	27.48 (1.70) ***	−4.31 (3.82)	3.17 (7.94)	1.39 (0.72)
**Exposure time**	−0.08 (0.05)	1.47 (1.70)	3.26 (2.61)	9.61 (5.42)	1.03 (0.41) *
**[CuO] × Time**			5.72 (1.40) ***	9.15 (2.90) **	−0.68 (0.22) **
***r*^2^**	0.37	0.85	0.72	0.74	0.20
***p* value**	3.31 × 10^−8^	2.2 × 10^−16^	2.36 × 10^−12^	5.41 × 10^−13^	0.0011
**F value**	21.86	131.6	38.42	42.13	5.92
**DF**	75	45	44	44	73

* *p* < 0.05; ** *p* < 0.01; *** *p* < 0.001.

## 4. Experimental Section

We purchased mussels from Taylor Shellfish Farms (Seattle, WA, USA) and kept them in flowing, sand-filtered seawater for one week prior to the start of experiments. We used a total of 80 mussels in the experiment. We chose mussels for our study based on total shell length (TL), which we measured using digital calipers (0.01 mm) along the longest axis of the shell. We placed individual mussels measuring 30 ± 1 mm into polyethylene cups containing 150 mL of 0.45 μm filtered seawater, aerated the containers, and kept them at 14 °C. We exposed mussels to CuO ENPs at 1, 2, and 3 mg L^−1^ for four weeks. We changed the water every other day, at which point 100 μL of food, concentrated phytoplankton (Shellfish Diet 1800, Reed Mariculture, Campbell, CA, USA), was added to each cup to obtain a phytoplankton concentration of 1.3 × 10^6^ cells mL^−1^.

We purchased CuO ENPs from Sigma-Aldrich (St. Louis, MO, USA) and previously characterized these ENPs [26]. CuO ENPs were found to be 84.8% ± 2.7% pure (impurities included Na, Ca, Si, and Mg) and 200–1000 nm in diameter. We prepared stock suspensions of 1 g CuO ENPs L^−1^ prior to each water change by adding ENPs to purified water (Barnstead Nanopure, Thermo Fisher Scientific, Waltham, MA, USA, resistivity ˃18 MΩcm) containing 500 mg L^−1^ of Suwannee River natural organic matter (International Humic Substances Society, St. Paul, MN, USA) and sonicated this suspension in a bath sonicator (Branson 2510, Branson Ultrasonics, Danbury, CT, USA) for 15 min. We then diluted this suspension to 100 mg L^−1^ using 0.45 μm filtered seawater and sonicated again for 15 min. We measured size of ENPs in this suspension immediately after sonication using dynamic light scattering (Nano ZS90, Malvern Instruments, Inc., Westborough, MA, USA). We then added the appropriate amount of this suspension to cups containing 0.45 μm filtered seawater and 100 μL of feed to obtain concentrations of 1, 2, or 3 mg L^−1^ and a final volume of 150 mL. New suspensions were prepared and added to cups every other day during the water change.

We measured clearance rate weekly of five mussels in each group by removing 1 mL from each cup immediately after feeding and every 20 min thereafter for 2 h. We estimated phytoplankton concentration in the samples by measuring *in vivo* chlorophyll fluorescence using a fluorometer (Model 7200-043, Turner Designs, Sunnyvale, CA, USA), which we related to cell count via haemocytometer counts, and calculated clearance rate for each individual by fitting an exponential function to the decrease in phytoplankton concentration over time. The same mussels were used throughout the experiment for clearance rate measurements.

We sampled three mussels and mussel biodeposits weekly from each group to measure accumulation and deposition of Cu. We measured TL and froze mussels after collection. To collect biodeposits we poured out the overlying water in each container, collected the biodeposits, allowed them to settle overnight, carefully drained off the supernatant and gently rinsed the biodeposits with purified water three times. We dissected and separated gill from the remaining tissue of thawed mussels and rinsed these tissue samples in purified water twice. Mussel biodeposits and tissue samples were lyophilized for 48 h. We then weighed the lyophilized samples to the nearest 0.1 mg, digested them in concentrated trace-metal-free HNO_3_ (Sigma-Aldrich, St. Louise, MO, USA) for 2 h at 60 °C followed by another 2 h at 80 °C in glass vials and diluted them to 10% acid using purified water. We then measured Cu content of tissue and biodeposit samples using inductively coupled plasma atomic emission spectroscopy (ICPAES, Thermo ICAP 6300, Thermo Fisher Scientific, Waltham, MA, USA). We ran blank and standard solutions every 10 samples. At the end of four weeks, we collected all remaining mussels and measured TL to determine growth rates.

We tested whether clearance rate, Cu content in tissue and biodeposits, and growth rate varied as a function of CuO ENP concentration using multiple ordinary least squares (OLS) regression models. CuO ENP concentration, exposure time, and their interaction were factors in all models. We predicted that exposure to CuO ENPs would decrease clearance rate, increase Cu loading in tissues and biodeposits, and decrease growth rate, and that these impacts would become more pronounced with increased exposure. To test these predictions we constructed multiple regression models for each physiological parameter as follows:

*Y* = α + β_1_*Conc* + β_2_*Time* + β_3_*ConcTime* + ε (1)

where *Y* is clearance rate, Cu content in tissue or biodeposits, or growth rate, α is the physiological rate or Cu content of the control group at the beginning of the experiment, *Conc* is the CuO ENP exposure concentration, *Time* is the exposure time in weeks, and ε is the variability not explained by the model. If the interaction term was not significant, we removed it and reran the model. We predicted that growth and clearance rate would decrease with increasing CuO ENP concentration and exposure time and that Cu in tissue and biodeposits would increase with increasing CuO ENP concentration and exposure time. For all regression models, we examined residual and quantile-quantile plots to ensure homogeneity of variance and normal distribution of data.

## 5. Conclusions

Our results suggest that CuO ENPs are much less toxic than Cu ions to marine mussels. However, mussels exposed to CuO ENPs excreted biodeposits containing high concentrations of Cu that could impact infaunal species that inhabit mussel beds and surrounding soft sediment communities. Additionally, mussels accumulated Cu when exposed to CuO ENPs, so may represent a transport mechanism for Cu ENPs in the coastal marine environment, especially through trophic transfer and exposure to mussel predators such as crabs, fish, and even humans. Our work demonstrates the potential for ecosystem level impacts as well as trophic transfer of ENPs and illustrates the need for further work to determine the impact of ENPs on marine coastal environments. 
